# Exploring gendered racism and the mental health of rural Black women

**DOI:** 10.3389/fpubh.2025.1525165

**Published:** 2025-02-18

**Authors:** Christina J. Ezemenaka, Wanda Martin Burton, Sharlene Newman

**Affiliations:** ^1^Department of Medicine, University of Mississippi Medical Center, Jackson, MS, United States; ^2^Capstone College of Nursing, University of Alabama, Tuscaloosa, AL, United States; ^3^Alabama Life Research Institute, The University of Alabama, Tuscaloosa, AL, United States

**Keywords:** rural, women, Black – African, microaggressions, mental health, depression, gendered racism, social determinants of health

## Abstract

**Background:**

Gendered racial microaggressions adversely impact Black college women’s mental health, but less is known about rural Black women’s mental health.

**Objective:**

Examine the association between gendered racism, depression, and psychological distress.

**Methods:**

This cross-sectional study included 200 rural Black women from the Southeastern United States. Gendered racism was assessed using the Gendered Racial Microaggressions scale (GRMS). Linear regression was used to assess GRM subscales, depression (PHQ-9) and psychological distress (K6) as outcomes.

**Results:**

Of the 200, 21.5% reported depression and 31% reported moderate psychological distress. Depression increased with increasing stress appraisal of gendered racism, *p* = 0.002.

**Conclusion:**

Gendered racial microaggressions impact the mental health of rural Black women.

## Introduction

Despite improvements, Black women in the United States (U.S.) continue to suffer from persistent health disparities. Compared to other women in the U.S., Black women experience excess mortality including shorter life expectancies, higher rates of maternal mortality, cardiovascular disease, and obesity (1). The persistent health disparities reflect the structural inequities Black women face and contribute to their mental health (1–3).

Approximately 18% of U.S. adults have been diagnosed with depression (4). Depression is a major risk factor for suicide and serves as both an individual and community health burden (5). As the leading cause of mental illness and disability, depression is twice as likely to occur in women than in men (6). While there are racial disparities in depressive disorder (6, 7), national depression rates specific to Black women are unclear. National data are reported separately based on race (White Americans and Black Americans) or gender (men and women). Thus, national depression rates in Black women are not ascertainable due to the structural mechanisms of data collection (1). Importantly, one recent study found that depression rates significantly worsened in Black Americans compared to their White counterparts after COVID-19 (7).

Despite its severity, depression is poorly recognized and undertreated due in part to the varying presentation of depressive symptoms (8). There are several hundred possible combinations of symptoms that meet criteria for depression, with depressed mood as the most common presentation. However, in Black women, common depressive symptoms include somatic symptoms, irritability, and present with comorbidities. The differences in the presentation of symptoms further complicate clinical diagnosis and may result in underdiagnosis for Black women (8).

Additionally, past research suggests that stigma leaves Black women, particularly in rural settings, unwilling to discuss mental health issues (9, 10). However, a recent health assessment (11) using a community-based participatory approach found that rural Black women were willing to provide information about mental health, citing it as a significant concern. This suggests that community attitudes toward mental health may be shifting (11).

### Black women’s mental health

Mental health is influenced by social and structural factors. According to the CDC, social determinants that increase the likelihood of poor mental health include experiencing interpersonal and institutional discrimination, and lack of access to education, housing, healthcare, employment, and economic opportunities (12). Other risk factors include adverse childhood experiences and other types of interpersonal violence and social isolation (12). Despite experiencing multiple risk factors and being overburdened with social inequities, mental health often goes under-reported in Black Americans (13, 14). A review of the literature revealed that underdiagnosis in Black women impacts prevalence rates (8, 14).

The mental health of Black women is also complicated by structural determinants of health, such as gendered racism (2). Gendered racism is defined as the complex oppression experienced by women of color based on the simultaneity of racism and sexism (15). Gendered racism often manifests as microaggressions. Gendered racial microaggressions (GRM) are subtle and daily verbal, behavioral, and environmental expressions of oppression based on the intersection of one’s race and gender (16). GRM has been related to a myriad of adverse mental health outcomes, including psychological distress and depression (16–21).

Though the mental health impact of experiencing GRM is clear, much of the extant literature is based on college samples and those with higher incomes (16–20). There is a dearth of literature on the relationship between GRM and the mental health of rural Black women in the Southeastern U.S. The purpose of this study is to explore the relationships between gendered racial microaggressions (GRM) and mental health within a rural community sample of Black women.

### Theoretical frameworks

To understand GRM, we draw from two theoretical frameworks, intersectionality and racial microaggressions. Intersectionality theory (22, 23) has been applied to public health equity research (24, 25). Intersectionality posits that women of color experience multiple, inseparable identities based on race and gender/sex that are tied to multiple marginalizations, such as racism and sexism. An intersectional approach uncovers the health outcomes of women of color made invisible by single-lens metrics that focus on race or gender. Rather, an intersectional lens to health understands that race and gender are inseparable, socially constructed through norms and policies, and vary based on other social identities (e.g., social class, ability, age, immigration history) (25). Thus, gendered racism provides a more complete view of Black women’s experiences of discrimination as it accounts for their gender and race, Gendered racism may be experienced as microaggressions (16). Racial microaggressions theory (26) suggests that subtle forms of oppression are normalized and stem from the idea that racism is normalized, woven into the fabric of American society, and may appear ordinary (15). Identity-based microaggressions include intentional and unintentional insults, putdowns, invalidations, slights, and offensive behaviors as well as through environmental policies and ideologies that cumulatively harm mental health (25).

Depression is severe yet underdiagnosed in Black women. Depressive symptoms often manifest differently in this population due to the influence of social and structural determinants of mental health (33). Absent research that specifically focuses on rural Black women and risk factors such as GRM, prevalence rates will remain unknown. This study aims to determine the prevalence of depression and psychological distress among Black women in rural Alabama and explore the relationship between depression and GRM while controlling for sociodemographic characteristics among Black women in rural Alabama. We also sought to explore the relationship between psychological distress and GRM while controlling for sociodemographic characteristics among Black women in rural Alabama.

## Methods

### Procedures

As part of a parent community-based participatory research (CBPR) study (11), community health surveys were administered in five rural Alabama communities. The purpose of the CBPR study was to assess health concerns among residents. The study was implemented in partnership with the Alabama Conference of Black Mayors. Community member representatives, selected by their respective mayors because of their knowledge and engagement in their communities, facilitated the data collection process. Convenience sampling was used to recruit participants through a variety of community events. Paper and pencil surveys were distributed at community events organized by the mayors of each community. The events included health fairs, vaccine clinics, and community education meetings. Surveys were also administered via canvassing the community to obtain a representative sample. Each representative completed an orientation process consisting of human subjects research training. Results of the parent study suggested a high prevalence of mental health concerns among residents. In consequence, an additional data-collection phase was added to further identify the mental health concerns of Black women. The University of Alabama’s institutional review board approved this study, protocol #21-03-4465.

### Setting and participants

Enrolled participants (*n* = 206) were self-identified Black women living in rural Alabama communities, aged 18 and older. Verbal consent was obtained from all participants before the study commencement.

### Eligibility criteria and analytic sample

Inclusion criteria involved self-identification of Black race, female gender, residence in a rural Alabama community among adults over the age of 18. Participants with completed surveys containing non-missing values for depression and psychological distress were eligible for study inclusion. Surveys with missing depression and psychological distress (n = 6) scores were excluded from the analyses. The final analytic sample included 200 participants.

### Instruments

The Gendered Racial Microaggressions Scale (GRMS) is a 26-item multidimensional validated scale that assesses the lifetime frequency and stress appraisal of gendered racial microaggressions (16). A sample item includes: “Someone accused me of being angry when I was speaking in a calm manner.” The GRMS frequency composite is scored using a 6-level response scale, ranging from 0 to 5 where 0 = “never,” 1 = “less than once a year,” 2 = “a few times a year,” 3 = “about once a month,” 4 = “a few times a month,” and 5 = “once a week or more.” The GRMS stress appraisal composite is scored using a 6-level response scale, ranging from 0 to 5 where 0 = “this has never happened to me,” 1 = “not at all stressful,” 2 = “slightly stressful,” 3 = “moderately stressful,” 4 = “very stressful,” and 5 = “extremely stressful.” For each composite, total scores are found by averaging the 26 items; this generates a scoring scale with a range of 0 to 5. GRMS consists of four subscales specific to Black women: beauty assumptions and sexual objectification, silenced and marginalized, Strong Black Woman, and Angry Black Woman. The GRMS frequency (*α* = 0.95) and stress appraisal (*α* = 0.97) composites had good internal consistency for this sample (16).

### Outcomes

The Patient Health Questionnaire (PHQ-9) assesses the severity of self-reported depressive symptoms over the past two weeks (27). We used eight of the nine questions, known as PHQ-8. The ninth item was deleted as it asks about suicidal attempts and ideation. Although DSM-IV criteria assess suicidal ideation, this item was deemed inappropriate for nonclinical research that lacks the ability to conduct an appropriate intervention (28). A sample item reads, “Over the past two weeks, how often have you been bothered by the following problems? Little interest or pleasure in doing things.” Response categories for each item are based on a 4-point Likert scale where 0 = “not at all,” 1 = “several days,” 2 = “more than half the days,” and 3 = “nearly every day.” Total scores are found by summing the eight items. For scores ≥10, the PHQ-8 has high sensitivity and specificity for depression (27). In this study, scores ≥10 indicate current depression. The PHQ-8 had good internal consistency for this sample (*α* = 0.91). Higher scores indicate increased depression severity.

The Kessler 6 (K6) is a six-item validated scale that measures non-specific psychological distress (PD) during the past 30 days (29). A sample survey question is, “During the past 30 days, about how often did you feel nervous?” Item responses are based on a 5-point Likert scale from 0 to 4 where 0 = “none of the time” and 5 = “all of the time” (21). Total scores are found by summing the scores from all six questions. Psychological distress scores range from 0 to 24 with scores from 0 to 4 classified as none or low mental distress, scores from 5 to 12 indicating moderate mental distress, and scores ≥13 indicating serious mental distress, possibly warranting further assessment (30). The K6 had good internal consistency for this sample (*α* = 0.86).

### Independent variables

The following sociodemographic characteristics were considered as independent variables in this study: age, education, income, and relationship status. For analysis purposes, we operationalized these variables as follows: age (continuous), education (some high school (reference); high school diploma or GED; some college or college degree), income [≤ $2,999 (reference); $25,000 to $49,999; ≥ $50,000], relationship status [married; not married (reference)]. GRMS frequency and stress appraisal composite scores were also included as covariates.

### Statistical analysis

Descriptive statistics were used to summarize characteristics of the cohort using means and standard deviations for continuous variables and frequencies and percentages for categorical characteristics. We used linear regression to independently examine the association between depression and psychological distress outcomes, GRMS frequency, and stress appraisal composite scores while controlling for age, education, income, and relationship status. We did not impute missing data. Participants missing values for outcome measures were excluded from the analyses. The significance level for comparisons was set at *p* ≤ 0.05. JMP Pro 18 was used for all analyses.

## Results

### Cohort summary

Participants (*N* = 200) were Black females and were, on average, 56.6 years (SD = 16.1). The majority (26.3%, *n* = 45) had completed high school, 21% reported earning a college degree, and 96.7% identified as non-Hispanic. Over half of the sample (51.2%) earned an income of less than $24,999. The relationship status reported by most participants (31.5%) was *married*; see Table 1.

**Table 1:** Summary statistics of study cohort.

Characteristic	*N* = 200
**Age, Mean (SD)**	56.6 (16.1)
Missing	20
Ethnicity, *N* (%)	
Hispanic	6.0 (3.3)
Non-Hispanic	176.0 (96.7)
Missing	18
Education, *N* (%)	
Some high school	38.0 (22.2)
High school diploma or GED	45.0 (26.3)
Some college	44.0 (25.7)
Associate’s degree	19.0 (11.1)
Bachelor’s degree	8.0 (4.7)
Graduate degree	17.0 (9.9)
Missing	29
Income, *N* (%)	
$15,000 or less	41.0 (25.6)
$15,000 to $24,999	41.0 (25.6)
$25,000 to $34,999	26.0 (16.3)
$35,000 to $49,999	30.0 (18.8)
$50,000 to $74,999	10.0 (6.3)
$75,000 to $99,999	3.0 (1.9)
$100,000 to $149,999	6.0 (3.8)
$150,000 to $199,999	2.0 (1.3)
$200,000 or over	1.0 (0.6)
Missing	40
Relationship status, *N* (%)	
Married	57.0 (31.5)
Never married	52.0 (28.7)
Widowed	40.0 (22.1)
Divorced	24.0 (13.3)
Separated	8.0 (4.4)
Missing	19
**Psychological distress, Mean (SD)**	4.5 (4.7)
**Depression, Mean (SD)**	6.0 (6.3)
Gendered Racial Microaggressions Scale, Mean (SD)	
Stress appraisal	1.3 (1.4)
Missing	73
Frequency	1.3 (1.2)
Missing	21

### Depression and psychological distress

In this sample, the mean depression score was 6.0 (SD = 6.3, Range: 0–24) with 43 (21.5%) reporting depression (PHQ-8 ≥ 10). For psychological distress, 13 (6.5%) reported serious (K6 ≥ 13), 62 (31%) reported moderate (K6: 5–12), and 125 (62.5%) reported none to low mental distress, with a mean of 4.5 (SD = 4.7, Range: 0–24), Table 1. The GRMS composite subscale of stress appraisal was positively correlated with depression, *r* (127) = 0.27, *p* = 0.002, but not with frequency (*p* = 0.237). As stress appraisal increases, depression increases. Neither GRMS frequency nor stress appraisal was significantly correlated with PD, all *p* > 0.05; see Table 2.

**Table 2:** Correlations of composite GRMS stress appraisal and frequency, depression, and psychological distress.

Variables	Depression	Psychological distress	GRMS frequency
GRMS frequency	0.09 (*n* = 179)	0.08 (*n* = 179)	-
GRMS stress appraisal	0.27** (*n* = 127)	−0.05 (*n* = 127)	0.75** (n = 121)

**p ≤ 0.01; *p ≤ 0.05.

GRMS frequency and stress appraisal were assessed in different models per outcome as they were highly correlated. Due to multicollinearity, income was not included in the regression models. The final regression models best explain the variation in depression, psychological distress, GRM, and demographic characteristics. Missingness among GRMS frequency and stress appraisal composite scores was evaluated in this sample. Neither were associated with model covariates nor outcomes, all *p* ≥ 0.05.

## Analysis results

### Depression

Depression was significantly associated with GRMS stress appraisal, [*F* (1, 125) = 10.1, *R*^2^ = 0.074, *p* = 0.002]. For every unit increase in stress appraisal, depression scores increased by 1.2 units; see Table 3. Models containing GRMS frequency and other covariates were not statistically significant, *p* > 0.05.

**Table 3:** Linear regression analysis of GRMS stress appraisal and depression.

Independent variable	Beta	95% CI^1^	*p*-value
Intercept	4.5	3.1, 6.1	<0.0001
GRMS stress appraisal	1.2	0.5, 2.0	0.002

^1CI, confidence interval. R2 = 0.074, N = 127.^

### Psychological distress

Psychological distress was significantly associated with education, but not with GRMS stress appraisal (*p* = 0.9212), [*F* (3, 104) = 2.7, *R*^2^ = 0.072, *p* = 0.0483]. Compared to those who completed some high school, participants with some college or a college degree saw a decrease of 3.5 units in psychological distress scores, and participants with a high school diploma or GED saw a decrease of 1.5 units; see Table 4. In a separate model, psychological distress was significantly associated with education, but not with GRMS frequency, age (*p* = 0.0779), or relationship status (*p* = 0.4055), [*F* (5, 136) = 2.5, *R*^2^ = 0.084, *p* = 0.0331]. Compared to those who completed some high school, participants with some college or a college degree saw a decrease of 3 units in psychological distress scores, and participants with a high school diploma or GED saw a decrease of 1.1 units; see Table 5.

**Table 4:** Linear regression analysis of GRMS stress appraisal, sociodemographic characteristics, and psychological distress.

Independent variable	Beta	95% CI^1^	*p*-value
Intercept	7.0	4.4, 9.6	<0.0001
GRMS stress appraisal	0.04	−0.7, 0.8	0.9212
Education (ref = Some high school)			
High school diploma or GED	−1.5	−4.35, 1.4	0.3167
Some college or college degree	−3.5	−6.1, −0.8	0.0103

^1CI, confidence interval. R2 = 0.072, N = 108.^

**Table 5:** Linear regression analysis of GRMS frequency, sociodemographic characteristics, and psychological distress.

Independent variable	Beta	95% CI^1^	*p*-value
Intercept	8.8	5.3, 12.4	<0.0001
GRMS frequency	0.2	−0.5, 0.9	0.5415
Education (ref = Some high school)			
High school diploma or GED	−1.1	−3.5, 1.4	0.3900
Some college or college degree	−3.0	−6.1, −0.8	0.0050

^1^CI, confidence interval. *R*^2^ = 0.084, *N* = 142. Model adjusted for age (*p* = 0.0779) and relationship status (*p* = 0.4055).

## Discussion

To explore whether GRM impacts rural Black women’s mental health, we first examined the rates of self-reported depression and psychological distress. In this sample, approximately 21.5% reported depression and approximately 38% reported moderate or serious psychological distress. While depression rates in this sample are similar to the average in the state of Alabama (24%) in 2020, the current findings add nuance to state and national-level data on depression. National and state prevalence tends to vary by age and race (5). Typically, younger adults (21.5%) have higher rates than older adults (14.2%), and White adults (21.9%) have higher rates than Black adults (16.2%) (5). Given this sample’s older average age of 56 years and race, one might expect lower depression rates as suggested by national trends. However, our findings suggest that older, Black women’s rate of depression is higher than expected.

In addition to depression, PD was also prevalent in this sample, with 31% indicating moderate PD. Moderate PD has been deemed clinically meaningful because it optimally identifies individuals needing mental health treatment. Further, those in the moderate category are at risk for substance use, sedentary behavior, and obesity further underscoring the link between mental and physical health (30).

Current findings suggest that GRM is associated with depression. The stressfulness of GRM accounted for 7.4% of the variance in depression. While GRM was not associated with psychological distress, our results indicate that rural Black women who have completed some college or have obtained a college degree have lower mental distress than those who completed some high school. Substantial evidence has been presented on education as a social determinant of health (1, 12) and our research supports the buffering impact of increased education on mental distress. These findings add to the growing body of literature that supports GRM’s association with adverse mental health outcomes (16–21, 31).

This study must be viewed in light of some limitations that could be addressed in future research. First, participant data was captured at a single point in time using a cross-sectional study design. Therefore, we were not able to assess how the relationship between GRM and mental health outcomes changes over time. Second, convenience sampling was used to enroll participants, resulting in sampling bias. Thus, our results may not be generalizable to Black women residing in non-rural counties or outside the state of Alabama. Third, our survey utilized self-report instruments, increasing the potential for response bias. Consequently, participant responses may not accurately reflect mental health dispositions. Finally, we did not ask participants about their personal experiences of gendered racism and their effect on overall well-being. Future studies should employ longitudinal designs with a qualitative component to track participant metrics in a comprehensive manner. Including participant narratives will help close the gap in our understanding of the complex relationship between mental health and experiences of gendered racial microaggressions in this population. In addition, simple random sampling methods and objective metrics for mental health outcomes may also be employed to enhance the generalizability of study results. Despite the limitations of our study, our findings add to the mental health literature on the structural inequities rural Black women face that may harm their mental health.

## Conclusion

According to the CDC, 18.4% of US adults reported having ever been diagnosed with depression. Furthermore, Alabama ranks in the top 10 states with the highest prevalence of depression (5). The prevalence rates of depression and psychological distress in this sample of rural Black women emphasize the importance of not only allocating resources to the areas of greatest need (5) but to do so using an intersectional approach to mental health interventions (32) to account for the inequities associated with gendered racism. While national data reports stratify mental health prevalence rates by age, ethnicity, gender, etc., the mental health prevalence of the intersection of these groups, such as Black women, is not presented with these results. This gap in the national data collection mechanism warrants the need for exploratory studies that aim to capture and describe the mental health of specific subgroups while considering how the social and structural determinants of health influence the well-being of the population. Results from such studies will help inform culturally sensitive interventions designed to impact these populations in a meaningful way.

## Data Availability

The raw data supporting the conclusions of this article will be made available by the authors, without undue reservation.
